# A nonlinear relationship of evoked responses following charge-balanced single-pulse electrical stimulation with varying pulse widths

**DOI:** 10.1016/j.brs.2026.103049

**Published:** 2026-02-05

**Authors:** Isabel A. Danstrom, Joshua A. Adkinson, Zoe Liu, Meghan E. Robinson, Denise Oswalt, Garrett P. Banks, Atul Maheshwari, Lu Lin, Ben Shofty, Mohammed Hasen, Alica Goldman, Eleonora Bartoli, Sarah R. Heilbronner, Kelly R. Bijanki

**Affiliations:** a Department of Neurology, Baylor College of Medicine, Houston, TX, USA; b Department of Neurosurgery, Baylor College of Medicine, Houston, TX, USA; c Department of Neuroscience, University of Minnesota Medical School, Minneapolis, MN, USA; d KBR Inc., Houston, TX, USA; e Department of Neurosurgery, Perelman School of Medicine, University of Pennsylvania, Philadelphia, PA, USA; f Department of Neuroscience, Baylor College of Medicine, Houston, TX, USA; g Department of Neurosurgery, Clinical Neurosciences Center, University of Utah Health, Salt Lake City, UT, USA; h Imam Abdulrahman Bin Faisal University, College of Medicine, Dammam, Saudi Arabia; i Department of Electrical and Computer Engineering and Center for Neuroengineering, Rice University, Houston, TX, USA; j Department of Psychiatry, Baylor College of Medicine, Houston, TX, USA

## Abstract

**Background:**

Single-pulse electrical stimulation (SPES) can help guide neuromodulation therapy in an iterative process to reveal ideal circuits and degrees of engagement. Understanding the relationship between parameter input and neural output will be necessary both to build informative models of the brain’s functional connectivity and to improve responses to stimulation-based neuromodulation therapies. Modulating pulse width alters the total charge delivered to neural tissue and is thought to selectively activate fibers with different diameters, potentially shifting therapeutic thresholds. The anterior cingulate cortex (ACC) and orbitofrontal cortex (OFC) are of great clinical relevance to the pathophysiology and treatment of neuropsychiatric disorders.

**Objective:**

To provide empirical evidence for the impact of pulse width on pulse-evoked responses and promote the ability to modify specific circuits.

**Methods:**

We measured evoked response robustness, peak amplitude, and latency to peak amplitude from depth electrode contacts in 20 epilepsy patients undergoing intracranial monitoring for treatment-refractory epilepsy.

**Results:**

A nonlinear relationship between pulse width with evoked potential robustness was determined. Pulse width modulation is further shown to be distance-dependent, with distant connections responding maximally to a shorter pulse width.

**Conclusion:**

These results emphasize the importance of input stimulation parameters on cerebro-cerebral evoked potential (CCEP) response magnitude and consistency and are a step towards guiding selective engagement of specific fiber populations for both research and clinical settings.

## 1. Introduction

Direct electrical stimulation of specific neural pathways can be used to target and treat a range of neurological and psychiatric disorders. The design of stimulation parameters can have a substantial impact on neural selectivity and energy efficiency and thus is important across clinical settings. The stimulation waveform can vary with respect to morphology, number of phases, polarity, intensity, pulse width, duration, frequency, and current versus voltage control [1]. Insufficient responses to stimulation-based therapies are partly due to inconsistencies in selected stimulation parameters compounded by a lack of understanding of input-output relationships [2]. Using single-pulse electrical stimulation (SPES) can help to unmask the influence of individual components.

SPES is a promising technique to aid in parameter optimization due to its ability to map in vivo functional connectivity and dynamic brain network organization. Although SPES was historically used to map epileptic networks and identify seizure onset zones [3–8], its utility has expanded into explorations of brain organization more generally, as well as furthering our understanding of the mechanisms of stimulation-based neurotherapeutics. SPES is conducted using a series of individual pulses, and evoked potentials are measured at sites local and distant to the stimulation electrode contact. In this way, SPES can elucidate causal influence between brain regions with high spatiotemporal resolution (millisecond, millimeter) [9,10].

Each parameter of a stimulation waveform contributes to the resultant neural output. Computational models as well as in vivo and in vitro experiments have aided in demystifying relationships between input parameters and neural responses [2,11–15]. Multi-compartment cable models suggest that orientation-specific selectivity can occur based on the selection of cathodic versus anodic stimulation [13]. Further, tuning the frequency of stimulation can induce neural suppression or excitation [16,17]. Also, increasing stimulation amplitude influences neuronal activation, affecting the magnitude of evoked responses and the volume of neural tissue activated (VTA) [18].

Of particular interest, modulating the duration of the stimulation pulse (pulse width) is known to influence neural responses by altering the VTA, influencing energy efficiency, and is thought to selectively engage axons of varying diameters [1,11,19]. In deep brain stimulation (DBS) for movement disorders, a pulse width of 60 μs has been conventionally used, though advancements in implanted pulse generators (IPGs) have since enabled evaluation of shorter pulse widths. Several studies have focused on investigating whether a shorter pulse width influences clinical efficacy in comparison to the conventional pulse width, with findings largely supporting that the shorter condition shifts the therapeutic window [13,20–22]. A recent review summarizes results across a multitude of Parkinson’s disease (PD) and essential tremor DBS studies, reporting that therapeutic windows relative to amplitude range widen with shorter pulse width, but not with respect to total charge per pulse [23]. A shorter pulse width also may reduce tremor symptoms in essential tremor patients. Conversely, longer pulse widths narrow the therapeutic window [24].

SPES studies offer a more flexible environment than DBS for evaluating the relationship of pulse width modulation with neural response, though current evidence is limited to a few studies. One study reported increased evoked response amplitudes following longer pulse widths [2]. However, when maintaining total charge per phase, shorter pulse widths with higher stimulation amplitudes evoked greater response amplitudes [3]. By contrast, in silico models suggest a nonlinear relationship between pulse width and engagement of varying axon diameters, in which a medium duration would evoke greater magnitude responses than shorter or longer conditions due to optimal chronaxie engagement [11]. Under this simulated environment, longer pulse widths preferentially engage small fibers, and short durations preferentially engage large fibers, whereas medium pulse widths may maximally engage both populations.

However, the complexity of the human brain, including region-specific anatomical and functional characteristics, makes characterization of the precise effects of pulse width tuning particularly challenging. Although ideally the intricate input-output relationships for SPES will eventually be understood brain-wide, defining this is both more urgent and more plausible for some regions than others [25–30]. The anterior cingulate cortex (ACC) is one of these high-priority regions. The ACC is central to the limbic system as well as to the pathophysiology of a host of psychiatric and neurological conditions, including depression, anxiety, schizophrenia, obsessive-compulsive disorder, and chronic pain [31–35]. Anatomically, the ACC is situated such that nearly all of its projections course in, near, or through the cingulum bundle (CB) [36–38]. The CB is comprised of both myelinated and unmyelinated axons derived from neuronal populations ranging from large-diameter pyramidal neurons to small-diameter neuromodulators and von Economo neurons [36,38–40]. SPES directed to the CB produces consistent cortico-cortical evoked potentials [41], and nonlinear dynamics in neural responses following frequency modulation have been previously reported during cingulate stimulation [12]. Together, the heterogeneity of the white matter network engaged by the ACC – paired with the advantages of the ACC as a SPES target – offers a model environment for investigating the effect of pulse width tuning on neural responses. Furthermore, growing evidence supports the ACC as a viable neuromodulation target for psychiatric and pain indications [32,33,42–44]. Generally, neuromodulation therapies for psychiatric disorders target white matter in an effort to impact entire brain networks [45–49].

In this paper, we investigate variability in evoked neural responses associated with pulse width tuning. We employed a biphasic, symmetric SPES paradigm across 3 charge-balanced conditions while modulating pulse width. We hypothesized that ACC stimulation would elicit robust evoked potentials varying as a function of the pulse width condition. In alignment with prior work showcasing nonlinear dynamics in neural selectivity in response to ACC stimulation [12] and computational modeling of pulse width tuning [11], we expected to observe non-linear responses, wherein a medium pulse width may result in the strongest evoked responses. One possible underlying mechanism is that the medium pulse width engages both small and large fiber populations.

## 2. Materials and methods

## 2.1. Human participants and recruitment

Informed consent was obtained from 20 patients (mean age: 36, range 20–68 years; 50% female) with medically intractable epilepsy undergoing stereo-encephalography (sEEG) at Baylor St. Luke’s Medical Center (BSLMC) (Houston, TX, USA) (Table 1). All experiments were conducted in accordance with the principles outlined in the Declaration of Helsinki and approved by the Institutional Review Board at Baylor College of Medicine (IRB: H-18112). Three patients had prior stimulation treatment (Vagus Nerve Stimulation (1), Responsive Neurostimulation (1), or Deep Brain Stimulation (1)), and one patient had a prior anterior temporal lobectomy.

### 2.2. Electrode localization

Patients underwent pre-operative, high-resolution magnetic resonance imaging (MRI) to acquire anatomical brain data used in electrode localization. Anatomical scans were performed at either the Core for Advanced Magnetic Resonance Imaging (CAMRI) (Houston, TX, USA) or at BSLMC. T1-weighted images were acquired on a Siemens Prisma 3T system (CAMRI) using a Magnetization-Prepared Rapid Acquisition Gradient Echo (MPRAGE) sequence (Repetition time (TR) = 2400 ms, Echo time (TE) = 2.24 ms, Flip angle = 8^◦^, FOV = 256, 208 slices, 0.8 mm isotropic voxels) or a Philips Ingenia 3T system (BSLMC) (TR = 22 ms, TE = 4.6 ms, flip angle = 25, 1 mm isotropic voxels). Following invasive surgical implantation of electrodes, computed tomography (CT) scans were acquired at BSLMC.

Intracranial electrode positions were determined using the intracranial Electrode Visualization software pipeline (iELVIS) [50]. Post-operative CT scans were co-registered to pre-operative T1-weighted anatomical scans using the linear image registration tool (FLIRT) from the Functional Magnetic Resonance Imaging for the Brain Software Library (FMRIB). BioImageSuite [51] was used to manually identify electrode contact locations. Cartesian coordinates from individual electrode contacts were extracted for utilization in labeling contacts relative to individual brain regions and represent the spatial location of the contact with respect to RAS (increasing right, anterior, and superior axes) imaging storage order. Each contact was assigned to a brain region according to a joined HCPex volumetric [52] (cortical and subcortical) with XTRACT probabilistic white matter atlas [53]. Coordinate locations of electrode contacts were assigned to cortical, subcortical, or prominent white matter tracts according to the joint HCPex-XTRACT atlas. In the case that an electrode contact existed outside of the atlas parcels, a 5 mm radius was applied to assign the contact to the region in nearest spatial proximity. This process was repeated for all patients.

### 2.3. Stimulation location selection

**Fig. 1.**
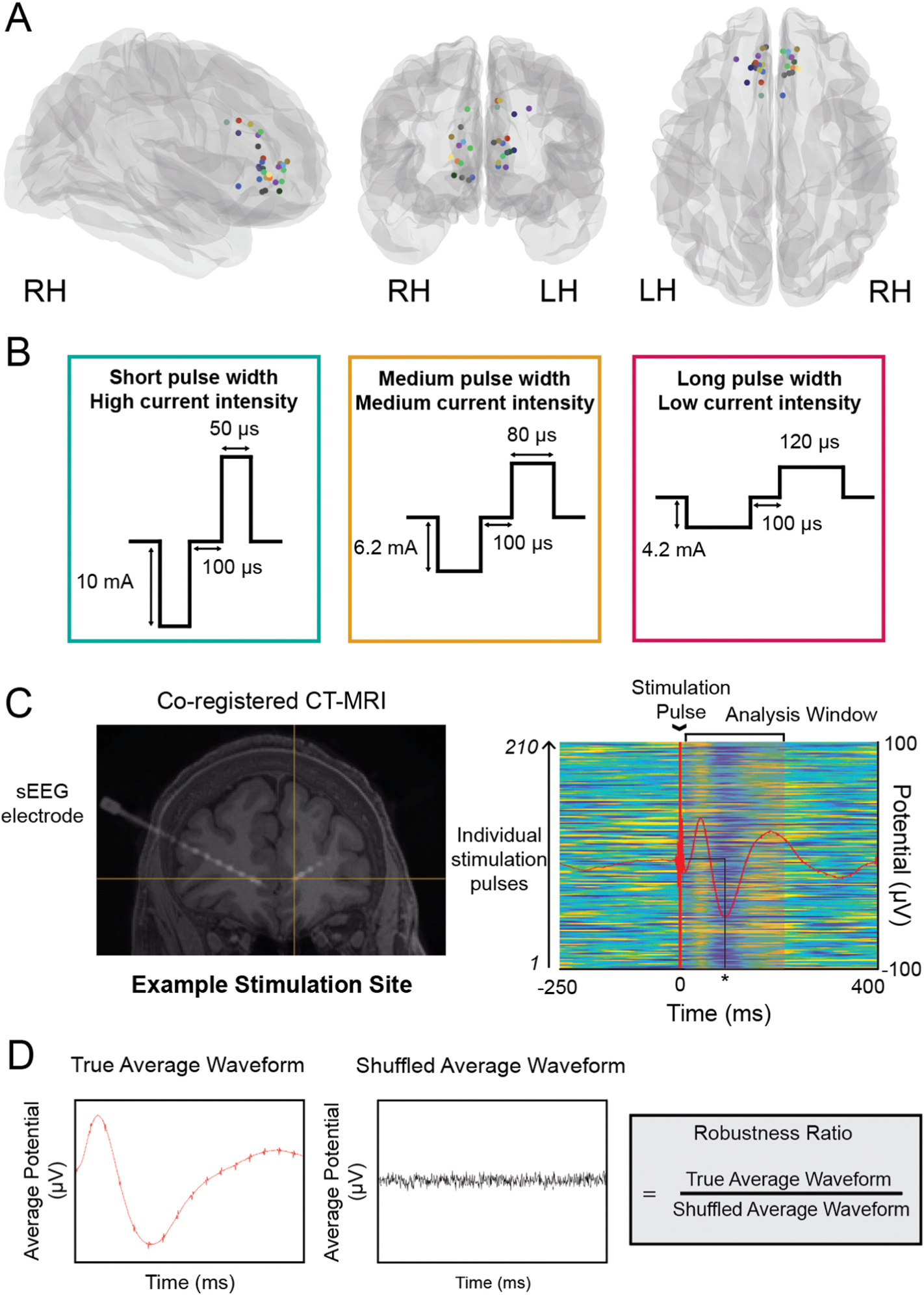
Single Pulse Electrical Stimulation (SPES) Experimental Design. **A.** ACC stimulation locations for all patients projected into 3-dimensional MNI305 space and visualized from sagittal, coronal, and axial views. Each color represents data from a different patient. **B.** Pulse width (PW) – Amplitude SPES conditions. **C.** (Left) Overlaid co-registered CT-MRI 2-dimensional coronal view of an example selected site. White areas within the brain are implanted sEEG electrodes (individual contacts appearing as spherical along the electrode probe). Crosshairs are centered on a selected stimulation site residing in ACC. (Right) Average evoked potential response in red underlaid by a heatmap of individual trials (left y-axis); voltage is shown on the right y-axis and time on the x-axis. The * is used to indicate the location in time where peak amplitude and latency is calculated from, depicting the voltage value (in μV) and time (in ms) where peak amplitude is reached between the 10–200 ms analysis window. **D.** Calculation of Robustness Ratio (RR) response metric. RR is calculated from the ratio of variance in the potential from the true average waveform relative to a time-decimated simulated average waveform.

For each patient, electrode contacts residing in or near the anterior cingulate cortex were identified and selected as stimulation sites (n = 29 stimulation sites, data from 16 patients) (Fig. 1A). Stimulation contacts were selected in a multi-step process. First, candidate stimulation contacts were identified using intra-operative overlaid CT with the pre-operative structural T1-weighted image. Then, contacts were confirmed to be localized to the ACC using the electrode localization pipeline, and then further inspected visually. Stimulation sites were also categorized as being within white matter, gray matter, or on the white-gray boundary using the electrode localization pipeline described above, along with visual confirmation on high-resolution anatomical scans. The Euclidean distance of each stimulation contact from the white-gray boundary was also computed. In addition to the ACC, stimulation was also delivered to the orbitofrontal cortex (OFC) (n = 5 sites, 4 patients) to evaluate the generalizability of pulse width tuning influence across other brain areas.

### 2.4. Single-pulse electrical stimulation experimental design

A monopolar stimulation paradigm using biphasic, cathode-first rectangular pulses was applied under three charge-equivalent pulse width (PW) conditions. Pulse width and current amplitude parameters were varied across conditions while holding total charge per phase constant at 0.5 μC: (1) short pulse width (50 μs, 10 mA), (2) medium pulse width (80 μs, 6.2 mA), and (3) long pulse width (120 μs, 4.2 mA) (Fig. 1B). An interphase interval of 100 μs was used between cathodic and anodic phases for efficiency and safety [54–56]. For each stimulation site and condition, 210 pulses were applied (Fig. 1C) with an inter-pulse period of 600 ms and a random jitter which ranged uniformly between 400 and 800 ms.

Due to clinical constraints, patients were implanted with depth electrodes from one of two manufacturers: (1) PMT Corporation (0.8 mm diameter; 8–16 contacts; 3.5 mm center-to-center distance; 0.0503 cm^2^ surface area) (PMT Corporation, MN, USA) or (2) AdTech Medical Instrument Corporation (1.28 mm diameter; 9 contacts; 5.0 mm center-to-center distance; 0.089 cm^2^ surface area) (AdTech Medical Instrument Corporation, WI, USA). Because electrode surface area differs across manufacturers, matching charge per phase across pulse width conditions results in different absolute charge densities (PMT: 9.86–10.82 μC/cm^2^; AdTech: 5.57–5.66 μC/cm^2^: range due to technical limitations of stimulation equipment). Charge density was therefore not independently controlled across stimulation electrode types at matched charge/phase. Importantly, all comparisons of the impact of pulse width condition on evoked responses are performed within stimulation-recording pairs, such that charge density remains consistent across pulse-width conditions for any given stimulation contact. Thus, the primary manipulation is how varying PW conditions differentially engage neural tissue.

During all experiments, adverse event monitoring was conducted constantly by epilepsy monitoring unit technicians, and researchers were in open communication with the attending epileptologist.

### 2.5. Stereo-electroencephalography (sEEG) recordings and signal processing

Local field potentials from electrode contact channels (n = 6388 stimulation-recording pairs) were recorded with a 256-channel Blackrock Cerebus System (Blackrock Microsystems, UT, USA) at a 30 kHz sampling rate and filtered with a high-pass 0.3 Hz first-order Butterworth and a low-pass 7500 Hz third-order Butterworth applied during acquisition. Signals were referenced during acquisition to a pre-selected reference electrode contact visually confirmed during the implant surgical procedure to be situated in deep white matter. Processing of signals was conducted using custom MATLAB software (written by J.A.). Prior to data acquisition, recording channels with poor signal quality were manually identified and tracked for removal from analysis. Signals from recording electrodes were evaluated using another custom MATLAB (J.A.) interface for the presence of stimulation artifact, interictal spiking, or line noise contamination and removed from further analysis upon detection. Additionally, to remove recording bias related to the extent of the stimulation field, channels immediately adjacent to the stimulation contact were ignored during analysis.

Cerebro-cerebral evoked potentials (CCEPs) were identified within a 10–200 ms post-stimulation window, capturing both direct and indirect responses, using a two-step thresholding approach. For each recording channel, the z-scored signal was taken relative to a pre-stimulation window (−120 to −20 ms) to account for variability in baseline noise. Candidate peaks were then assessed based on meeting two requisite conditions: exhibiting a minimum prominence of 15 μV in the raw voltage trace and corresponding to local extremum in the z-scored trace exceeding a threshold of 6 standard deviations from baseline (positive or negative). Channels meeting these criteria were retained for subsequent analyses. Voltage recordings from the analysis window were averaged across the 210 trials to create a recorded average potential for each channel. The absolute value of the trial-averaged maximum voltage response was calculated and extracted as the Peak Amplitude. A simulated average potential was then calculated by permuting trials in time for 1000 iterations to eliminate any time domain-based signal characteristics consistent across trials while keeping all original voltage values and generating a channel-specific noise waveform (Fig. 1D). At each iteration, the ratio of the standard deviation of the recorded average potential was compared to that of the simulated average potential, and the average ratio across iterations was computed as the robustness ratio (RR). RR thus reflects both the strength and consistency of the evoked response [54]. Peak amplitude, latency to peak amplitude, and RR were extracted for every recording electrode at each pulse width stimulation condition.

### 2.6. Statistical analysis

Statistical analyses were organized around a primary hypothesis that pulse width modulates evoked response magnitude under charge-equivalent stimulation, followed by secondary analyses testing whether this effect was modified by distance, stimulation location, or response timing. Non-parametric tests were used for condition-wise comparisons, and linear mixed-effects modeling was used to evaluate interactions. Exploratory analyses are explicitly labeled as such. Non-parametric statistical methods were employed due to non-normal data distributions (Shapiro Wilk results: Short PW: W = 0.857, *p* < 0.0001, Medium PW: W = 0.913, *p* < 0.0001, Long PW: W = 0.895, *p* < 0.0001). Friedman rank sum tests (χ^2^) followed by post-hoc Nemenyi tests (Q) were conducted to compare CCEP response magnitude and consistency across PW stimulation conditions. For comparisons across multiple groups and non-repeated measures, Kruskal-Wallis tests (H) were used with subsequent post-hoc tests (Dunn’s test, Z) for pairwise comparisons. For comparing proportions, chi-square test for given probabilities and two-sample tests for equality of proportions were used. All statistical tests were corrected for multiple comparisons when necessary, using Bonferroni, single-step, or continuity correction method. Effect sizes for statistical tests are included in Supplementary Table 1.

To evaluate the influence of pulse width and anatomical distance on evoked responses, we fit linear mixed-effects models using the lmer function in R (lme4 package). Robustness Ratio (RR) and latency to peak amplitude were modeled as dependent variables, with fixed effects for pulse width condition (50 μs, 80 μs, and 120 μs), Euclidean distance from the stimulation site, and their interaction. Random intercepts were included for unique stimulation-recording pairs or subjects, as appropriate. Models were fit using restricted maximum likelihood (REML), and p-values for fixed effects were computed using Satterthwaite’s approximation (lmerTest package). Coefficients (β), standard errors, t-values, degrees of freedom, and p-values are reported alongside calculated confidence intervals obtained using profile likelihood. The anova (.) function (lmerTest package) was used to assess omnibus significance for main effects and interactions when necessary. Model comparisons were performed using likelihood ratio tests and AIC/BIC criteria. P-values equal or larger than *p* = 0.0001 are reported exactly, while p-values smaller are reported as *p* < 0.0001.

## 3. Results

SPES was performed to assess the impact of pulse width modulation on evoked neural responses. We stimulated the frontal cortex in 20 patients and recorded neural activity from intracranial depth electrodes distributed densely across frontal, parietal, and temporal lobes (Supplementary Fig. 1). There were no patient reports of behavioral effects from any of the stimulation sites, and no clinical or electrographic seizures were induced by the stimulation. We focused on stimulation of the anterior cingulate cortex (ACC) and the orbitofrontal cortex (OFC).

### 3.1. Evoked responses to stimulation exhibit a nonlinear pattern across pulse width conditions

**Fig. 2.**
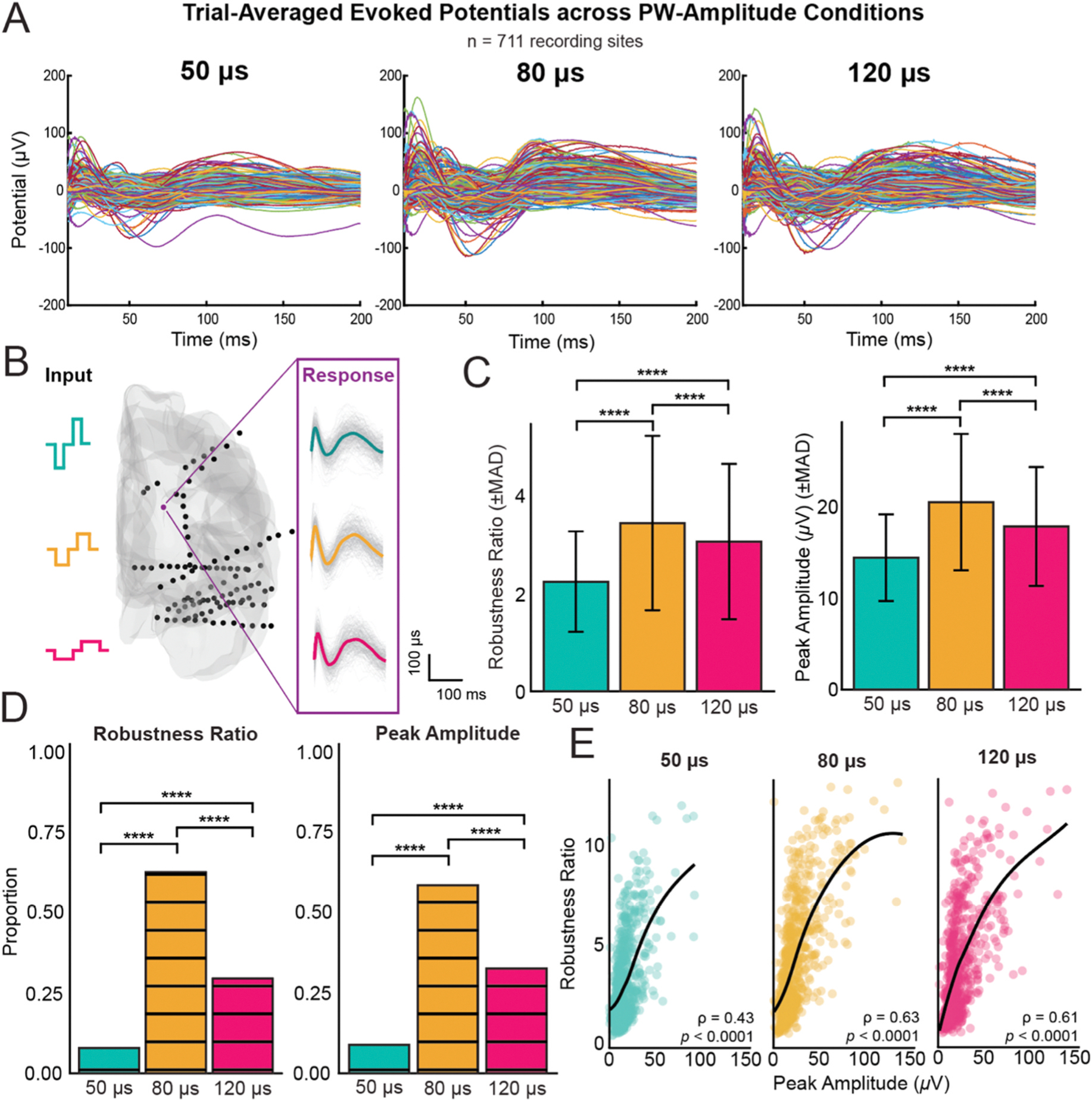
Evoked potential responses across pulse width conditions. **A.** Trial-averaged neural traces across recording sites (n = 711 channels) for each PW condition. **B.** Input stimulation waveforms with exemplar trial-level (n = 210 per condition, gray traces) and overlaid trial-averaged (colored by condition) neural traces across short, medium, and long PW stimulation conditions. **C.** Median Robustness Ratios (RRs) (left) and Peak Amplitude (right) across all recording sites (n = 711) for patients following stimulation at short (50 μs), medium (80 μs), or long (120 μs) pulse width stimulation (Median Absolute Deviation (MAD)). **D.** Proportion of recording sites with strongest RR responses to the different PW conditions (RR: 50 μs: n = 56, 80 μs: n = 445, 120 μs: n = 210; Peak Amplitude: 50 μs: n = 63, 80 μs: n = 416, 120 μs: n = 232). **E.** Spearman rank correlations among evoked potential features across stimulation conditions. Loess fit overlaid with black trace. (****p < 0.0001).

We first tested whether pulse width modulation under charge-equivalent stimulation produces systematic differences in evoked responses to anterior cingulate cortex (ACC) stimulation. We applied SPES at 3 pulse width conditions while balancing the overall charge per phase by modulating stimulation amplitude and calculated the evoked response robustness and peak amplitude for each level (n = 711 stimulation-recording pairs, n = 29 stimulation contacts, n = 16 patients). Trial-averaged traces from all responses across each stimulation condition are displayed in Fig. 2A. Trial-by-trial responses from an example channel are presented with anatomical reference in Fig. 2B. Compared with the short or long pulse width, the medium condition resulted in significantly stronger evoked potentials to ACC stimulation with respect to both response robustness and amplitude (Robustness Ratio (RR): χ^2^ = 502.3, p < 0.0001; Short vs. Medium: Q = 31.1 p < 0.0001; Short vs. Long: Q = 20.7, p = 0.0001; Medium vs. Long: Q = 10.4, p = 0.0001) (Peak Amplitude: χ^2^ = 422.2, p < 0.0001; Short vs. Medium: Q = 28.4, p < 0.0001; Short vs. Long: Q = 19.7, p < 0.0001; Medium vs. Long: Q = 8.66, p < 0.0001) (Fig. 2C). This nonlinear effect of pulse width tuning was highly consistent across both stimulation site and patient levels (Supplementary Fig. 2). Further, we found that this effect was specific to stimulation and recording sites that demonstrated a structural connection, measured using deterministic tractography (Supplementary Fig. 3). These results were additionally confirmed using linear mixed effects modeling of RR with PW condition as a fixed effect, with a random effect for stimulation-recording pair to account for repeated measures (Supplementary Table 2). Given that some patients received stimulation from different electrode types, we conducted a control analysis evaluating the impact of this variable (Supplementary Fig. 4). The amplitude of the evoked responses at the 80 μs condition was modulated by the electrode type, which we explored further by running a mixed effects model, incorporating electrode type as a random effect (Supplementary Table 3). Indeed, the electrode type varied across the sample, and it was possible that the effect was actually explained by inter-individual variability rather than electrode type itself. Indeed, the addition of electrode type to the mixed-effect model exerted no influence on the detected nonlinear pattern in evoked responses.

Recording channels were next binned according to which pulse width induced the strongest response, representing the stimulation condition a channel was maximally responsive to. All 711 channels were retained in this analysis. Most of the electrode pairs exhibited a preference for the medium pulse width paradigm compared to the long or short condition (RR: χ^2^ = 323.9, p < 0.0001; Short vs Medium: *p* < 0.0001, Short vs Long: p < 0.0001, Medium vs Long: *p* < 0.0001) (Peak Amplitude: χ^2^ = 263.1, p < 0.0001; Short vs Medium: *p* < 0.0001, Short vs Long: p < 0.0001, Medium vs Long: *p* < 0.0001) (Fig. 2D). These results highlight that while many channels display a maximized response to the 80 μs stimulation condition, other channels have the largest response to either the 50 μs or 120 μs condition. Lastly, we evaluated the correlation among evoked response features at each pulse width condition and found a consistent strong positive relationship (50 μs: ρ = 0.429, *p* < 0.0001; 80 μs: ρ = 0.629, *p* < 0.0001; 120 μs: ρ = 0.619, *p* < 0.0001) (Fig. 2E, Supplementary Fig. 5). These results establish a robust, non-linear effect of pulse width on evoked responses, with maximal responses at the intermediate pulse width.

### 3.2. Spatial distribution of evoked responses across pulse width stimulation conditions

**Fig. 3.**
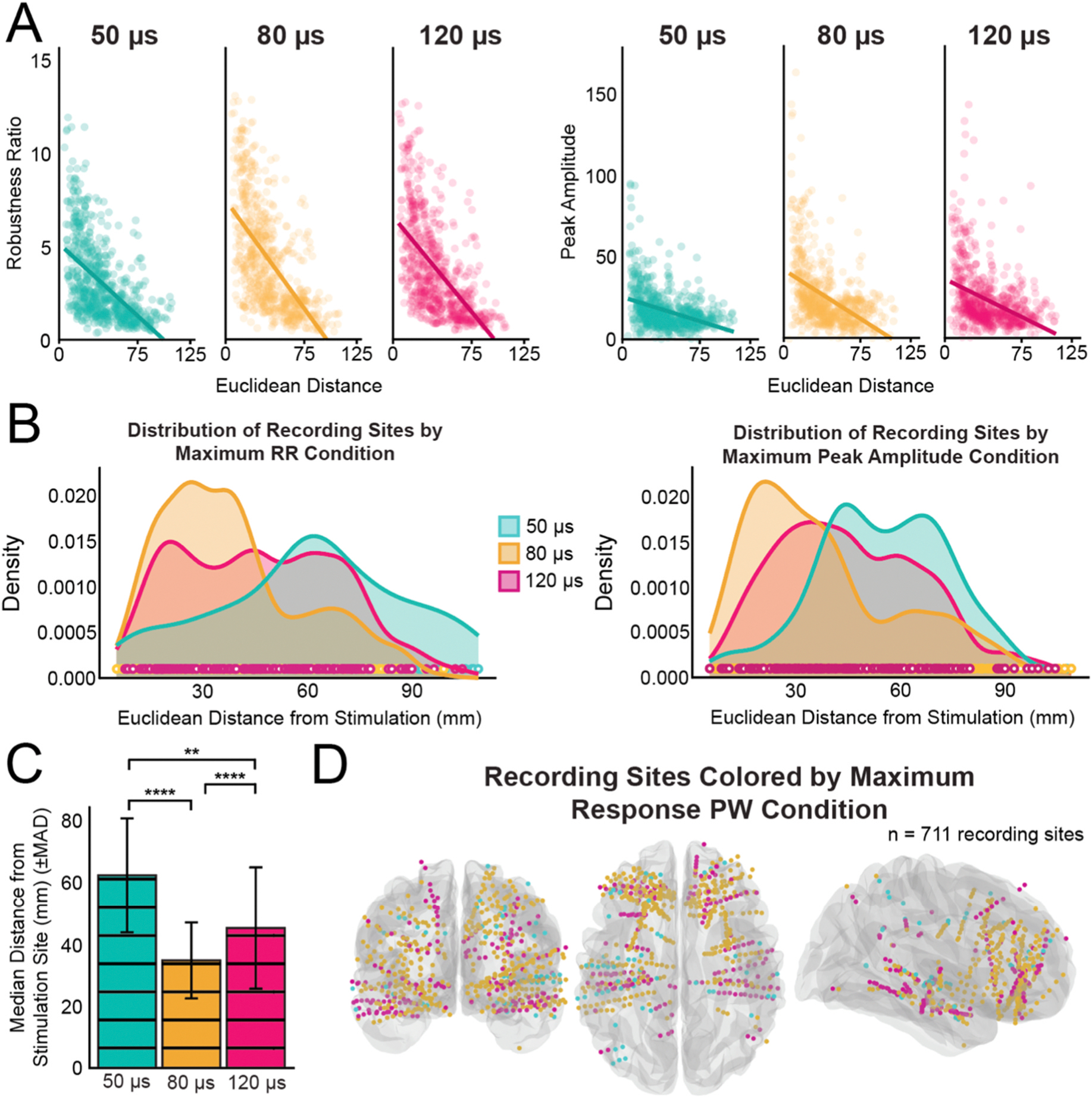
Interaction of Euclidean distance with pulse width modulation. **A.** Distributions of RR (left) and Peak Amplitude (right) across Euclidean distance for each PW condition. **B.** Density distribution of recording channels sorted based on maximum response condition for either RR or Peak Amplitude (condition which evoked strongest RR or largest amplitude) (RR: 50 μs: n = 56 recording sites, 80 μs: n = 445, 120 μs: n = 210; Peak Amplitude: 50 μs: n = 63 recording sites, 80 μs: n = 416, 120 μs: n = 232) **C.** Median Euclidean distance between recording site and the stimulation site following separation of recording sites by maximum RR PW condition. **D.** Aggregation of recording sites colored by preferred PW condition. (**p < 0.01, ****p < 0.0001) (RH = right hemisphere, LH = left hemisphere). Error bars reflect MAD.

We next interrogated the relationship of the center-to-center Euclidean distance between stimulation and recording sites with stimulation condition and evoked response robustness. Using a linear mixed-effects model with a random intercept for unique stimulation-recording contact pair, we evaluated the effect and interaction of distance and pulse width on evoked responses (both RR and Peak Amplitude). Across conditions, both RR and Peak Amplitude decreased with Euclidean distance (RR: β = −0.0769 ± 0.0034 per mm, t = −22.6, 95% CI [−0.0836, −0.0702], *p* < 0.0001; Peak Amplitude: β = −0.396 ± 0.026 per mm, t = 15.5, 95% CI [−0.447, −0.346], *p* < 0.0001), and the finding that maximal responses are produced following stimulation at the 80 μs condition was confirmed (RR: 50 μs: β = −2.32 ± 0.077, t = −30.0, 95% CI [−2.47, −2.17], *p* < 0.0001; 120 μs: β = −0.855 ± 0.077, t = −11.1, 95% CI [−1.01, −0.704], *p* < 0.0001; Peak Amplitude: 50 μs: β = 16.6 ± 0.77, t = −21.6, 95% CI [−18.13, −15.11], *p* < 0.0001; 120 μs: β = −5.57 ± 0.77, t = −7.22, 95% CI [−7.08, −4.06], *p* < 0.0001) (Fig. 3A). We found that the interaction term was also significant for both features (RR: F (2,1418) = 132.4, *p* < 0.0001; Peak Amplitude: F (2,1418) = 79.91, *p* < 0.0001), indicating that the slope of RR across distance differs by pulse width. Compared to the 80 μs condition, the negative slope of distance was most attenuated for the short PW condition (RR: β = 0.0258 ± 0.00161, t = 16.1, 95% CI [0.0227, 0.0290], *p* < 0.0001; Peak Amplitude: β = 0.202 ± 0.016, t = 12.6, 95% CI [0.171, 0.233], *p* < 0.0001), and less so for the long condition (β = 0.009342 ± 0.00161, t = 5.81, 95% CI [0.00619, 0.0125], *p* < 0.0001; Peak Amplitude: β = 0.089 ± 0.016, t = 5.56, 95% CI [0.058, 0.120], *p* < 0.0001). Taken together, these results indicate that the effect of distance on evoked responses is different across pulse width conditions, and that shorter pulses exhibit the shallowest decay, becoming relatively more effective at longer distances (within the range of recorded distances in the dataset).

To further probe the differential effect of distance across pulse width conditions and provide descriptive context, the median Euclidean distance was compared between the stimulation and recording electrode contacts for pairs sorted by the pulse width condition producing the largest RR and amplitude (Fig. 3B). Recording sites with the largest response robustness to the short pulse width were significantly further from the ACC compared to sites preferring the medium or long conditions (Fig. 3C) (H = 45.2, *p* < 0.0001; Short vs Medium: Z = −5.93, *p* < 0.0001; Short vs Long: Z = −3.23, *p* = 0.00376; Medium vs Long: Z = 4.24, *p* < 0.0001). These results were consistent when sorting recording channels based on the PW condition evoking largest amplitude response (H = = 47.5, *p* <0.0001; Short vs Medium: Z = −5.97, *p* <0.0001; Short vs Long: Z = −2.99, *p* = 0.00841; Medium vs Long: Z = 4.67, *p* < 0.0001). Localized position of recording channels is shown in Fig. 3D, and each channel is colored by the PW condition producing the largest RR. These results suggest that the effect of pulse width is distance-dependent rather than region-specific. A list of included evaluated regions can be found in Supplementary Table 4. These findings were additionally conserved when estimating distance between stimulation and recording ROI using average streamline length from a normative population (Supplementary Fig. 6).

### 3.3. Generalization of pulse width effects beyond ACC

**Fig. 4.**
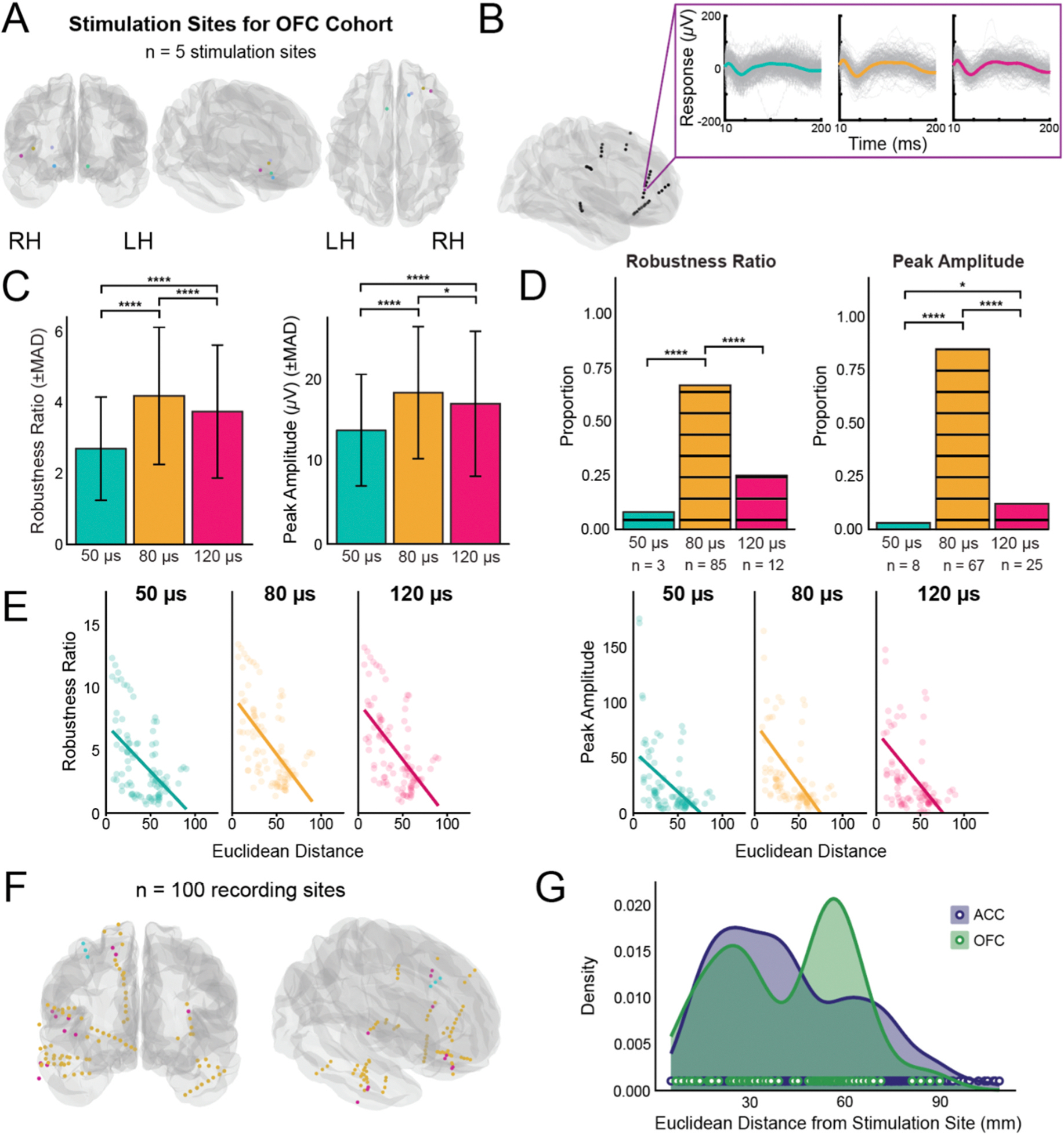
The effect of pulse width tuning during stimulation to orbitofrontal cortex (OFC). **A.** Anatomical location of stimulation contacts in the OFC (n = 5 stimulation sites, 4 patients). **B.** Exemplar neural trace for recording channel in the ipsilateral ACC following stimulation in the OFC, with trial-by-trial traces in gray overlaid by trial-averaged traces for each PW condition (50 μs in teal, 80 μs in golden yellow, and 120 μs in dark pink). **C.** Median RR and Peak Amplitude following stimulation at each PW condition for OFC stimulation. **D.** Proportion of recording sites with highest RR or largest Peak Amplitude at each PW condition for OFC stimulation (n represents number of recording sites in each group). **E.** Evoked responses (left: RR, right: Peak Amplitude) of recording sites across Euclidean distance for each PW condition. **F.** Anatomical locations of recording channels, colored by PW condition producing largest RR. **G.** Distribution of recording sites for ACC and OFC cohort, with individual distances for recording channels along the bottom of the x-axis for visual reference. (*p < 0.05, ****p < 0.0001) (RH = right hemisphere, LH = left hemisphere). Error bars reflect MAD.

To test whether the nonlinear pulse width effect was specific to ACC circuitry, we repeated the analysis in a limited sample of OFC stimulation sites. Electrodes in the orbitofrontal cortex (n = 5 stimulation sites, 4 patients, Fig. 4A) were stimulated while recording intracranial neural responses under the same 3 pulse width conditions. Subsequent analyses were restricted to ipsilateral connections, and evoked potentials were detected using the same approach as in the ACC stimulation analysis. An exemplar trace response to OFC stimulation is shown in Fig. 4B in the context of all recording channels from one patient included in the analysis. Median response amplitude and robustness (n = 100 stimulation-recording site pairs) to OFC stimulation were largest at the medium pulse width condition (80 μs), and showed a graded decrease in potential magnitude following stimulation at long (120 μs) and short (50 μs) (χ^2^ = 78.706, *p* < 0.0001; Short vs Medium: Q = 12.369, *p* < 0.0001; Short vs Long: Q = 8.004, *p* < 0.0001, Medium vs Long: Q = 4.366, *p* = 0.0057) (Fig. 4C). Similarly to the ACC, a significantly larger proportion of recording sites displayed the maximal responses to the medium condition (RR: χ^2^ = 121.3, *p* < 0.0001; Short vs Medium: *p* < 0.0001; Short vs Long: *p* = 0.12; Medium vs Long: *p* < 0.0001; Peak Amplitude: χ^2^ = 55.3, *p* < 0.0001; Short vs Medium: *p* < 0.0001; Short vs Long: *p* = 0.0016; Medium vs Long: *p* < 0.0001) (Fig. 4D). From the OFC cohort, only 3% of recording channels displayed maximal RR for the short PW condition, and 8% had the largest amplitude response for the short condition.

The distance-dependent modulation of evoked response features across PW conditions observed in the ACC was also replicated in the OFC cohort (Fig. 4E and F). A significant distance by PW condition interaction (RR: F (2,196) = 19.6, *p* < 0.0001, Peak Amplitude: F (2,196) = 24.1, *p* < 0.0001) demonstrated that evoked responses decayed least for the short condition (RR: β = 0.0192 ± 0.00335, t = 5.72, 95% CI [0.01263, 0.02570], *p* < 0.0001; Peak Amplitude: β = 0.361 ± 0.0530, t = 6.81, 95% CI [0.258, 0.465], *p* < 0.0001). The interaction between high pulse width condition and distance was significantly shallower for amplitude (β = 0.117 ± 0.0530, t = 2.22, 95% CI [0.0141, 0.221], *p* = 0.0279), but not for RR (β = 0.00217 ± 0.00335, t = 0.647, 95% CI [−0.00437, 0.00870], *p* = 0.519). These interactions reinforce that shorter pulses (within the tested range) lose less signal magnitude and robustness with distance, yielding relative efficiency gains at longer ranges. To provide context relative to the ACC cohort, we compared distributions of Euclidean distance across OFC stimulation sites and ACC stimulation sites (Fig. 4G). A Mann-Whitney *U* test demonstrated that the distributions of Euclidean distance were not significantly different for the ACC stimulation cohort compared to the OFC cohort (W = 35534, *p* = 0.9944), indicating that the distances examined for both cohorts were similar. These results suggest that the nonlinear pulse width effect is not unique to ACC, though sample size limits stronger inferences.

### 3.4. Latency to peak amplitude responses following ACC or OFC stimulation

**Fig. 5.**
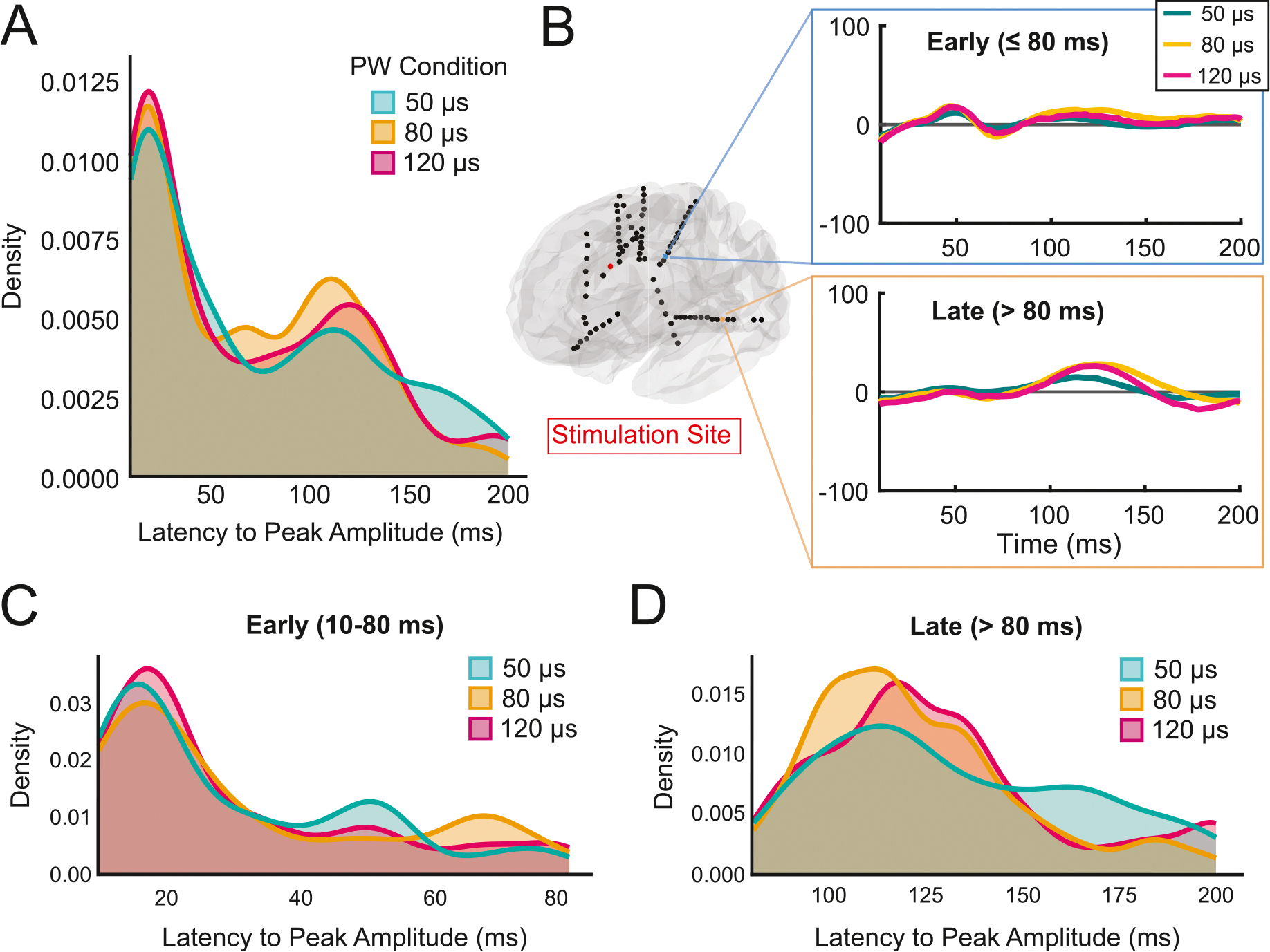
Relationship between latency to peak amplitude and pulse width modulation. **A.** Distribution of latency (in ms) to peak amplitude (absolute value of maximum positive or negative deflection taken between 10–200 ms) across all recording sites (n = 811) following stimulation at each PW condition. **B.** Example of a patient’s distribution of localized recording channels demonstrating evoked potentials in response to ACC stimulation, with the stimulation contact highlighted in red, and two recording channels displaying early (peak amplitude occurring at or before 80 ms) and late (peak amplitude occurring after 80 ms) responses. Trial-averaged traces are plotted across each PW condition for both recording channels. The recording channel demonstrating an early response is in blue, and the late in sandy brown. **C.** Density plot of latency distributions separated by PW condition for the early response window. **D.** Density distribution of responses occurring in the late window, separated by PW condition.

We examined latency to peak amplitude as a secondary feature to assess whether pulse width effects may reflect changes in response timing. To explore the potential relationship between the latency component of evoked responses and pulse width modulation, the distribution of latency to the maximum peak amplitude from the evoked waveform was evaluated in response to stimulation to the ACC and OFC (n = 34 stimulation sites, 19 patients, 811 recording channels). Peak amplitude was defined as the maximum potential (positive or negative deflecting) recorded between the 10–200 ms temporal window after delivery of the stimulation pulse, as described in the Methods. Response distributions are first shown across all channels and PW conditions (Fig. 5A). Response windows (early or late) were assigned to each individual recording channel based on when in the analysis window the peak amplitude response was detected, as CCEPs have distinct early and late responses [55]. We assigned recording channels to either early (≤80 ms) or late (>80 ms) response windows, motivated to integrate the window in which the maximum amplitude was recorded into the analysis. Example traces for early and late responses are provided in Fig. 5B. We used a linear mixed-effects approach to model latency to peak amplitude as a continuous variable with random intercepts for stimulation-recording pairs. Euclidean distance from the stimulation site was included as a centered covariate.

Unsurprisingly, a significant main effect of response window (early or late) was detected, with responses in the late group demonstrating significantly longer latencies compared to those in the early group (β = 95.47 ± 1.55, t = 61.6, 95% CI [92.4,98.5], *p* < 0.0001). PW condition alone did not significantly impact latency in the early phase (50 μs: β = −1.43 ± 1.22, t = −1.18, 95% CI [−3.80, 0.947], *p* = 0.240; 120 μs: β = −1.77 ± 1.120, t = −1.48, 95% CI [−4.12, 0.579], *p* = 0.140) (Fig. 5C). However, in the late phase, a significant effect was detected: responses at the short PW had longer latencies compared to the medium condition by approximately 10 ms (β = 9.95 ± 1.97, t = 5.05, 95% CI [6.09, 13.8], *p* < 0.0001) (Fig. 5D). Responses to the long PW were also delayed relative to the medium PW by about 6.5 ms (β = 6.47 ± 1.96, t = 3.31, 95% CI [2.64 10.3], *p* = 0.000963). Euclidean distance was positively related with latency in both the early and late phase, supporting longer latency is often associated with more distant recording positions (early: β = 0.121 ± 0.0356, t = 3.40, 95% CI [0.0510, 0.190], *p* = 0.000704; late x Euclidean distance interaction: β = 0.114 ± 0.0460, t = 2.47, 95% CI [0.0234, 0.204], *p* = 0.0135). These results were confirmed from estimated marginal means (at mean Euclidean distance). Latencies across channels were similar for each PW condition (50 μs: 29 ms, 95% CI [26.9, 31.1]; 80 μs: 30.5 ms, 95% CI [28.3, 32.6] 120 μs: 28.7 ms, 95% CI [26.6, 30.8], all *p* > 0.3). In the late phase, latencies were significantly longer following stimulation at the short PW condition (130.6 ms) (95% CI [132, 136.9] relative to either the medium (125.9 ms) (95% CI [123.4, 128.4], *p* < 0.0001) or long (95% CI [128.1, 133.1], *p* = 0.0273). These results demonstrate that changing the pulse width of the stimulation waveform modulates latency to peak amplitude predominantly in the late response phase (after 80 ms). Further, the 50 μs stimulation condition associated with longer latencies.

## 4. Discussion

As neuromodulation continues to expand its utility as a therapy for neuropsychiatric conditions, more foundational evidence substantiating the influence of specific stimulation parameters on neural responses is necessary. Building on accumulating data supporting fiber diameter-based selectivity [1,11], we investigated the pulse width-dependent modulation of evoked potential responses to SPES. We measured intracranial evoked response robustness and latency to peak response across 3 pulse width stimulation conditions applied to the ACC. Central tendencies in evoked responses revealed a graded, nonlinear pattern in the effect of pulse width tuning on ipsilateral connections. The 80 μs pulse width resulted in the strongest responses, in terms of both response robustness and amplitude, compared to the shorter or longer widths tested. Further, the highest proportion of recording sites displayed a preference for the 80 μs condition. A small collection of sites displayed preferences for the 50 μs or the 120 μs pulse width conditions. To further explore what may underlie these preferences, we investigated distance and latency as contributing factors. We found that at sites distal to the stimulation area, the shortest pulse width exhibited consistently stronger responses than the other paradigms, and that the reduction in response across distance is attenuated in the short condition. Finally, we observed latency-related modulation of responses across PW conditions, specifically for peak responses occurring late in the temporal window (>80 ms after stimulation). These results were consistent across different stimulated regions (ACC and OFC) and speak to the importance of optimizing stimulation parameters in terms of neural selectivity and to the correspondence of functional effective connectivity with anatomical connectivity.

Nonlinear stimulus-response relationships regulate multiple essential processes in the human brain [56,57]. Direct electrical stimulation introduces an invasive stimulus that disrupts neural systems to reveal linear or nonlinear stimulus-response relationships. We found a nonlinear response between ACC stimulation pulse width and neural response robustness. Other studies investigating the influence of pulse width modulation using SPES have found mixed results, with one study reporting increasing local response amplitude with a longer pulse width [2], another suggesting a linear, negative correlation (decreasing pulse width evoked stronger responses) [3], and a third finding no impact [58]. Clear distinctions between the studies (including the present study) exist, such as the inclusion of stimulations at subdural electrodes, monopolar versus bipolar contact configuration, or indiscrimination of stimulated brain regions. Any mix of these differences could result in contrasting findings, highlighting the need for further systematic studies on the effects of stimulation parameters across different circuits and stimulation setups.

We observed that the mid-level pulse width evoked the strongest response robustness, an effect that may be explained by differential engagement of fiber populations, though this was not directly tested in the current study. Computational modeling suggests that altering the waveform pulse width modifies the activation of axons varying in diameter, with shorter durations preferencing large fibers and long pulses engaging small diameter axons [11]. In alignment with biophysical hypotheses [19], we believe that in the conditions evaluated, the medium pulse width could be engaging multiple fiber diameters. Other, plausible explanations for the observed response could include maximal orthograde and retrograde circuit engagement of fibers at the medium pulse width, reduced engagement of inhibitory circuits, reduced variability in evoked potential responses across trials, or multiple evoked responses within the evaluated temporal window. Importantly, the premise that specific populations of neurons can be selectively targeted or avoided by parameter tuning is essential to optimizing invasive neuromodulation treatments. Engaging some, but not other, fibers may widen therapeutic windows by avoiding fibers related to side effects while explicitly activating therapeutic populations [21,22,59]. This sort of selective targeting to maximize clinical efficacy has been demonstrated through modeling stimulation fields in DBS for PD [15,60,61].

Selective activation of axons of specific diameters could also allow for targeting specific neuronal connections or subtypes. The ACC is a clinically relevant brain area serving roles in the manifestation, perpetuation, and treatment of neurological and psychiatric conditions, including depression, anxiety, obsessive-compulsive disorder, and neuropathic pain [32–35,42,43,62–64]. Identifying and distinctly engaging therapeutic fibers remains an ongoing goal of ACC-targeted brain stimulation [65–67]. Stimulation-induced neural selectivity has been previously shown to correspond to size, orientation, tissue type, and distance-based preferences [1,60] (Supplementary Fig. 7). The heterogeneity of cell type and axon diameters likely engaged from ACC stimulation [36,44,68–70] makes selective targeting a conceptually feasible objective.

We also identified an important interaction between pulse width modulation and distance from the stimulation site. The relationship of increasing distance with weakening evoked response is well-established [2,71]. In accordance, we found this decay evident across all tested pulse width conditions. However, the slopes significantly differed, suggesting that the effect of distance on evoked responses varied with respect to pulse width. The spatial distribution of recording site preference provides insight into underlying anatomical organization preferences. Specifically, long-range connections consistently responded maximally to the shortest pulse width. Long-range connections are more often mediated by axons with larger diameters [72,73], which promote rapid transmission of information due to fast conduction velocities [73,74]. Thus, in general, shorter pulse widths likely engage large-diameter fibers traveling long distances. Notably, there was a collection of short pulse width-preferring recording sites that were closer to the stimulation site. Deep fascicles contribute, although minimally, to cortico-cortical, intra-hemispheric connectivity [75]. The majority of these connections are bolstered by more superficial fibers, such as the U-fiber system, that connect adjacent cortical regions [73,76,77]. U-fiber systems contain axons ranging in inner diameters from 0.16 μm to 9 μm. Thus, a small proportion of large-diameter axons mediate shorter-distance connections, likely explaining their preference toward a short pulse width despite being proximal to the stimulation area.

Biophysical considerations may provide a parsimonious explanation for the observed nonlinear relationship between pulse width and evoked response robustness under charge-equivalent stimulation. Prior modeling work has shown that, when charge per phase is held constant, intermediate pulse widths can maximize axonal recruitment by balancing the activation thresholds of fiber populations with different diameters and membrane time constants. Particularly, studies of pulse-width tuning suggest that short pulse widths preferentially activate large-diameter axons, whereas longer pulse widths lower thresholds for smaller-diameter fibers, with intermediate pulse widths producing broader recruitment across fiber classes [11,78]. Given the short interphase delay used in the present study (100 μs), our biphasic waveforms are expected to approximate the recruitment patterns predicted for monophasic stimulation in these models. While we did not perform subject-specific field modeling or tract-integrated simulations, the consistency of the intermediate pulse-width preference across stimulation sites and patients is compatible with these established biophysical principles. Importantly, we emphasize that such models provide qualitative intuition rather than definitive mechanistic inference, and future work integrating patient-specific anatomy, tract geometry, and stimulation field modeling will be necessary to directly link pulse-width tuning to selective fiber recruitment in vivo.

While the interaction of pulse width and distance was discernible at the short pulse width condition, the same was not true of the proposed relationship among longer pulse width, shorter connections, and smaller diameter axons [11]. We would have expected to observe the average distance of contacts with maximal responses to the long pulse width condition to be shorter than that of mid-level pulse width. Instead, we observed a nonsignificant difference between the 80 μs and 120 μs conditions. One explanation could tie to the selected spectrum of pulse widths examined: perhaps an even longer pulse width would demonstrate this effect.

The nonlinear effect of pulse width tuning was conserved across a small sample of OFC stimulation sites. Responses to neural stimulation are sensitive to and dependent on the region stimulated, and sometimes even the functional domain within a given region [2,12,28]. The ACC and OFC share vast reciprocal connections and are both integral components of the limbic network [40,79]. As such, we cannot rule out that our findings are specific to areas involved in emotional processing or to frontal areas. Future work should focus on identifying variability in neural response dynamics to parameter modulation across regions and functional networks. Importantly, our data do not support that the medium pulse width finding is specific to the ACC, as the nonlinear CCEP response as well as distance-modulated response decay patterns were replicated in the OFC stimulation cohort. Notably, the OFC cohort was limited to 4 patients. Thus, while the nonlinear response pattern is consistent across stimulated regions, some properties may be region specific, necessitating follow-up investigations with larger cohorts.

There are several caveats related to our experimental design. We acknowledge the potential influence of underlying pathology and anti-seizure medications on the stimulation-response relationship. However, variability in these clinical factors was substantial across participants, and the consistency of response trends observed across patients reduces the likelihood that our findings are driven solely by disease-related factors. Notably, results from the OFC closely mirrored those observed in the ACC, further strengthening our primary conclusions. We emphasize that isolating the effects of any single stimulation parameter on evoked potentials requires holding the stimulated region (Supplementary Fig. 8) – and as many additional variables as possible – constant.

We also acknowledge limitations associated with the use of HCP-based tractography and recognize that integration of patient-specific neuroimaging data would offer important advantages for interpreting electrophysiological findings. In addition, we elected to utilize non-parametric statistical tests to examine the initial effect of PW condition on CCEP features. This decision was motivated by the absence of improved model fit when including subject-level random effects, and the non-parametric approach was deemed an appropriate alternative given potential violation of linear model assumptions.

Due to safety constraints of stimulation hardware, time limitations, and the need to maintain balanced charge across conditions for use in multiple patient cohorts, the maximum pulse width tested was 120 μs. We recognize that our total charge/phase (0.5 μC/phase) is slightly lower than commonly reported in biphasic CCEP studies [10,80]. Importantly, these parameters were sufficient to elicit robust and reliable CCEPs [81]. Future work should examine a broader range of pulse widths within established safety limits to increase sensitivity, as the full complexity of the pulse width-response relationship remains incompletely characterized.

Finally, two clinical depth electrode types with different contact surface areas were used, resulting in differences in absolute charge density across manufacturers. Control analyses indicated that electrode type did not contribute substantial additional variance to the observed effects (Supplementary Table 2), and the repeated measures design and statistical approach controlled for baseline heterogeneity between channels. Nevertheless, the present findings do not directly address how absolute charge density differences across electrode designs may influence response magnitude or spatial spread. Given the hypothesized role of charge density in shaping stimulation fields [82,83], future studies should explicitly characterize both charge density and total charge per phase.

This work demonstrates how optimizing the parameter space may enable selective modulation of structurally connected recording sites at varying distances and likely axon caliber. Therefore, depending on the goal of stimulation, we offer the following suggestions: for engaging the highest proportion of sites residing proximal to or mid-length from the ACC, employing a middle-range pulse width may best achieve this goal. Meanwhile, if the goal of intervention is to target long-range connections, a short duration may be preferred. Further research of charge-balanced pulse width modulation in the clinical space is necessary to better understand the association between pulse width and behavioral responses.

## 5. Conclusion

Efforts to achieve beneficial therapeutic responses to neuromodulation therapies have often outpaced an in-depth mechanistic understanding of stimulation effects. This work, along with many others, has made efforts towards closing the gap by exploring key components of the parameter space and uncovering relationships with neural output. Our results provide evidence for a nonlinear relationship of pulse width with evoked responses following ACC or OFC stimulation, with the highest proportion of recording sites displaying more robust responses to 80 μs pulse width compared to either 50 or 120 μs conditions (at 0.5 μC total charge per phase). These results, taken with the observed distance interaction in the ACC, underscore the importance of parameter tuning for selective network engagement and inform strategies for targeted neuromodulation and more focused stimulation mapping.

## Supplementary Material

1

## Figures and Tables

**Table 1:** Patient demographics.

Patient ID	Electrode Coverage	Sex	Age Range

1	RL	F	30–39
2	RL	F	40–49
3	RL	F	40–49
4	RL	F	40–49
5	RL	M	30–39
6	RL	M	40–49
7	RL	M	20–29
8	RL	F	40–49
9	RL	M	20–29
10	RL	F	20–29
11	RL	M	20–29
12	RL	F	20–29
13	RL	M	20–29
14	RL	M	50–59
15	RL	M	60–69
16	RL	F	30–39
17	RL	F	40–49
18	RL	M	20–29
19	RL	M	30–39
20	RL	F	40–49

RL = Right and Left hemisphere.

## Appendix A. Supplementary data

Supplementary data to this article can be found online at https://doi.org/10.1016/j.brs.2026.103049.
