# Supplementary material for: A nonlinear relationship of evoked responses following charge-balanced single-pulse electrical stimulation with varying pulse widths
Source: Brain Stimul. Author manuscript; Available in PMC 2026 Aug 5. (PMC13440464; doi:10.1016/j.brs.2026.103049)
Supplement: Supplementary file 1 [file NIHMS2190655-supplement-1.docx]

**Supplementary Material**

**Distribution of evaluated and selected recording channels for CCEPs.**

Recording sites forming ipsilateral connections with ACC stimulation sites across all patients (n = 16) are shown in Panel A, representing all candidate recording channels from the ACC cohort. Panel B shows the anatomical locations of channels where a CCEP was detected following stimulation to either the ACC or OFC.

***Supplementary Figure 1. Spatial distribution of recording channels*. (A).** Distribution of all recording sites from the ACC stimulation cohort of patients (n = 16). All recording sites formed ipsilateral connections with the stimulation site. **(B).** Distribution of recording sites with detected CCEPs following stimulation to the ACC or the OFC (n = 20).

**Supplementary Table 1. Calculated effect sizes for statistical tests included in the main manuscript.** Effect sizes for Friedman tests (Kendall’s W), Nemenyi tests (rank-biserial correlation (rbc)), Kruskal Wallis tests (epsilon squared (ε^2^)), or Dunn’s test (Cliff’s Delta (𝛿)) are included with respect to the matching figure panel.

| **Matching Figure Panel** | **Statistical Test Run** | **Test Statistic** | **p-value** | **Effect Size Test** | **Effect Size** |
| --- | --- | --- | --- | --- | --- |
| 2C (RR) | Friedman | 502.3 | < 0.0001 | Kendall’s W | 0.927 |
|  | Post-hoc Nemenyi Tests | | | | |
|  | 50 µs vs. 80 µs | 31.1 | < 0.0001 | rbc | -0.873 |
|  | 50 µs vs. 120 µs | 20.7 | < 0.0001 | rbc | -0.766 |
|  | 80 µs vs. 120 µs | 10.4 | < 0.0001 | rbc | 0.496 |
| 2C (Amp) | Friedman | 0.899 | 0.638 | Kendall’s W | 0.00258 |
|  | Post-hoc Nemenyi Tests | | | | |
|  | 50 µs vs. 80 µs | 28.4 | < 0.0001 | rbc | -0.771 |
|  | 50 µs vs. 120 µs | 19.7 | < 0.0001 | rbc | -0.659 |
|  | 80 µs vs. 120 µs | 8.66 | < 0.0001 | rbc | 0.260 |
| 3C | Kruskal-Wallis | 45.2 | < 0.0001 | ε^2^ | 0.0637 |
|  | Post-hoc Dunn’s Tests | | | | |
|  | 50 µs vs. 80 µs | -5.93 | < 0.0001 | 𝛿 | 0.475 |
|  | 50 µs vs. 120 µs | -3.23 | 0.00375 | 𝛿 | 0.302 |
|  | 80 µs vs. 120 µs | 4.24 | < 0.0001 | 𝛿 | -0.208 |
| 4C (RR) | Friedman Test | 148.5 | < 0.0001 | Kendall’s W | 0.957 |
|  | Post-hoc Nemenyi Test | | | | |
|  | 50 µs vs. 80 µs | 17.2 | < 0.0001 | rbc | -0.981 |
|  | 50 µs vs. 120 µs | 9.5 | < 0.0001 | rbc | -0.947 |
|  | 80 µs vs. 120 µs | 7.7 | < 0.0001 | rbc | 0.87 |
| 4C (Amp) | Friedman | 0.899 | 0.638 | Kendall’s W | 0.904 |
|  | Post-hoc Nemenyi Tests | | | | |
|  | 50 µs vs. 80 µs | 12.4 | < 0.0001 | rbc | -0.981 |
|  | 50 µs vs. 120 µs | 8.0 | < 0.0001 | rbc | -0.947 |
|  | 80 µs vs. 120 µs | 4.4 | < 0.0053 | rbc | 0.87 |

**Supplementary Table 2. Linear mixed-effects modeling of Robustness Ratio across PW conditions.** In addition to use of the non-parametric Friedman test (Figure 2, main manuscript), we also evaluated the impact of PW condition on Robustness Ratio using an LME modeling approach *(RobustnessRatio ~ PWCondition + (1 | Subject) + (1|Stimulation-Recording pair))*. Random effects for both Subject and unique Stimulation-Recording pairs were used. A total of 711 stimulation-recording pairs were included. The Friedman test was used as a suitable alternative for this modeling as the primary approach for two interacting reasons: not all model assumptions were met, and the statistical importance of accounting for the hierarchical nature of the data was found to be minimal (inclusion of Subject term did not significantly improve model fit).

| **Random Effects** | | | |
| --- | --- | --- | --- |
| **Groups** | **Name** | **Variance** | **Std. Dev.** |
| Stim-Rec Pair | (Intercept) | 3.61 | 1.90 |
| Subject | (Intercept) | 2.09 | 1.45 |
| Residual |  | 0.552 | 0.743 |

| **Fixed Effects** | | | | | |
| --- | --- | --- | --- | --- | --- |
|  | **Estimate** | **Std. Error** | **df** | **t-value** | **p-value** |
| (Intercept) | 3.97 | 0.379 | 15.6 | 10.5 | < 0.0001 |
| 50 µs | -1.22 | 0.0394 | 1420 | -30.9 | < 0.0001 |
| 120 µs | -0.458 | 0.0394 | 1420 | -11.6 | < 0.0001 |

^Reference group used was medium PW (80 µs)^

| **Estimated Marginal Means** | | | | | | |
| --- | --- | --- | --- | --- | --- | --- |
| **Contrast** | **Estimate** | **SE** | **df** | **t-ratio** | **p_adjusted_** |  |
| 80 µs and 50 µs | 1.219 | 0.0394 | 1420 | 30.9 | < 0.0001 |  |
| 80 µs and 120 µs | 0.458 |  |  | 11.6 | < 0.0001 |  |
| 50 µs and 120 µs | -0.761 |  |  | -19.3 | < 0.0001 |  |

^Tukey method used for p-value adjustment^

**Supplementary Table 3. Linear mixed-effects modeling of CCEP Responses across PW conditions with a random effect for stimulation electrode type.** In the present study, some patients were stimulated (ACC) with a different electrode type (AdTech: n = 12, PMT: n = 4), leading to different total charge density per phase (AdTech: 5.57-5.66 µC/cm²/phase; PMT: 9.86-10.02 µC/cm²/phase). Given that stimulation electrode type was not systematically assigned and was not the focus of inference, we designed a novel LME analysis incorporating this variable as a random effect to model it as a source of variance to control for heterogeneity in charge density/electrode properties. We found that results reported in Figure 2C of the main manuscript were unchanged with the inclusion of stimulation electrode type as a random effect. We additionally conducted Likelihood Ratio Tests for both Robustness Ratio and Peak Amplitude to evaluate the inclusion of the stimulation electrode type random effect. These results demonstrated no significant improvement in model fit with the addition of stimulation electrode type. Based on the Likelihood Ratio Tests, we found no evidence that stimulation electrode type explained additional variance beyond other sources of inter-individual and inter-electrode variability.

*RR ~ PWAmp + (1|StimElecType) + (1|SubjectID) + (1|Stim-Rec Pair)*

| **Random Effects** | | | |
| --- | --- | --- | --- |
| **Groups** | **Name** | **Variance** | **Std. Dev.** |
| Stim-Rec Pair | (Intercept) | 3.54 | 1.88 |
| SubjectID | (Intercept) | 1.88 | 1.37 |
| StimElecType | (Intercept) | 0.665 | 0.816 |
| Residual |  | 0.552 | 0.743 |

| **Fixed Effects** | | | | | |
| --- | --- | --- | --- | --- | --- |
|  | **Estimate** | **Std. Error** | **df** | **t-value** | **p-value** |
| (Intercept) | 4.17 | 0.658 | 0.938 | 6.35 | 0.11 |
| 50 µs | -1.22 | 0.0394 | 1220 | -30.9 | < 0.0001 |
| 120 µs | -0.458 | 0.0394 | 1420 | -11.6 | < 0.0001 |

^Reference group used was medium PW (80 µs)^

| **Estimated Marginal Means** | | | | | |
| --- | --- | --- | --- | --- | --- |
| **Contrast** | **Estimate** | **SE** | **df** | **t-ratio** | **p_adjusted_** |
| 80 µs and 50 µs | 1.22 | 0.0394 | 1420 | 30.9 | < 0.0001 |
| 80 µs and 120 µs | 0.458 | 0.0394 | 1420 | 11.6 | < 0.0001 |
| 50 µs and 120 µs | -0.761 | 0.0394 | 1420 | -19.3 | < 0.0001 |

^Tukey method used for p-value adjustment^

**Likelihood Ratio Test**

*Robustness Ratio*

Model 1: *RR ~ PWAmp + (1|SubjectID) + (1|Stim-Rec Pair)*

Model 2: *RR ~ PWAmp + (1|StimElecType) + (1|SubjectID) + (1|Stim-Rec Pair)*

| **LRT Results: RR** | | | | | | | |
| --- | --- | --- | --- | --- | --- | --- | --- |
| **Model** | **n_parameters_** | **AIC** | **BIC** | **LogLikelihood** | **Deviance** | **χ^2^** | **p_adjusted_** |
| Model 1 | 6 | 7002 | 7035.9 | -3495.0 | 6990 | 0.355 | 0.551 |
| Model 2 | 7 | 7003.6 | 7043.3 | -3494.8 | 6989.6 |  |  |

*Peak Amplitude*

Model 1: *PeakAmp~ PWAmp + (1|SubjectID) + (1|Stim-Rec Pair)*

Model 2: *PeakAmp~ PWAmp + (1|StimElecType) + (1|SubjectID) + (1|Stim-Rec Pair)*

*^** note, when executed, Model 2 returns as a singular fit^*

| **LRT Results: Peak Amplitude** | | | | | | | |
| --- | --- | --- | --- | --- | --- | --- | --- |
| **Model** | **n_parameters_** | **AIC** | **BIC** | **LogLikelihood** | **Deviance** | **χ^2^** | **p_adjusted_** |
| Model 1 | 6 | 16350 | 16384 | -8169 | 16338 | 0 | 1 |
| Model 2 | 7 | 16352 | 16392 | -8169 | 16338 |  |  |

**CCEP Responses across PW conditions for individual stimulation sites.**

A total of 29 stimulation sites were included in the analysis of ACC CCEP responses to varying PW conditions. Both RR and Peak Amplitude responses are depicted below at the level of individual stimulation contacts. Subject order presented here does not correspond with the order of subjects in main text Table 1, for the purpose of maintaining patient anonymity. The reported nonlinear trend in responses (RR and Peak Amplitude) across PW conditions is seen to be highly consistent (12/16 patients and 19/29 stimulation contacts), though not unanimous across the stimulation contact level. Each subject is colored by the electrode type of the stimulating electrode, either AdTech or PMT). No subject was stimulated at both electrode types.

***Supplementary Figure 2.*** ***RR and Peak Amplitude responses across individual recording sites*.** (*Left*) Median (underlying bar plot) and individual recording channel (individual line plots) RR responses sorted by stimulation contact. Stimulation contacts are further grouped by subject and colored by the stimulation electrode type (blue = PMT, orange = AdTech). *(Right)* Peak Amplitude voltage responses at the median and individual recording channel levels sorted by stimulation contact across all subjects.

**Weaker stimulation-evoked connectivity corresponds with the absence of structural connectivity across pulse width conditions.**

We used ROI assignments to predict the presence of structural connectivity between stimulating and recording sites (shown in Supplementary Figure 1). Tractography was conducted in 10 control participants from the Human Connectome Project 3T Young Adult diffusion MRI database using Anatomically Constrained Tractography in MRTrix3(Smith et al., 2012) for each ROI pair and the path distance of the connection was computed from the produced streamlines. We acknowledge the limitations of not using patient-specific diffusion data but unfortunately did not have pre-operative diffusion imaging data for all patients. For ROI pairs lacking a structural connection, no path distance was resolved. We sorted ipsilateral connections following ACC or OFC stimulation and included all channels where a CCEP was detected (n = 811 channels) since most non-structurally connected pairs were captured in the excluded cluster of data. Electrode contact pairs were separated based on the presence or absence of structural connectivity. Under each pulse condition, significantly higher RRs were recorded across structurally connected compared to pairs lacking a structural connection (Short: W = 3144, *p* < 0.05; Medium: W = 988, *p* < 0.0001; Long: W = 1045, *p* < 0.0001).

***Supplementary Figure 3.*** ***Evoked responses and structural connectivity.*** Structural connectivity between stimulation and recording site pairs was evaluated using diffusion-based tractography and the presence of absence of streamlines. Median robustness ratios were compared when splitting contact pairs by their connection status for each PW condition. (* p < 0.05, **** p < 0.0001).

**CCEP Responses across different Stimulation Electrode Types.**

Across patients, two types of depth electrodes were used. AdTech Medical Instrument Corporation electrodes (1.28 mm diameter; surface area 0.089 cm²) and PMT Corporation electrodes (0.8 mm diameter; surface area 0.0503 cm²) necessarily resulted in different absolute charge densities when charge per phase was matched across pulse-width conditions (AdTech: 5.57-5.66 µC/cm²/phase; PMT: 9.86-10.02 µC/cm²/phase). To investigate whether charge density could impact PW comparisons, recording sites were stratified by electrode manufacturer, and median responses were assessed at each PW level for AdTech (n = 414 recording channels stimulated on an AdTech electrode) and PMT (n = 297). Wilcoxon rank-sum tests indicated no significant differences in peak amplitude between stimulation electrode types at short (W = 60065, *p* = 0.601) or long conditions (W = 63643, *p* = 0.423), though peak amplitude responses to stimulation at PMT electrodes did demonstrate a higher amplitude response under the medium condition (W = 47295, *p* < 0.0001). However, given that repeated measures were accounted for at the individual stimulation-recording pair level, it is unlikely that this difference is responsible for the observed effects, especially since responses were compared using a within-contact design. Of note, this analysis does not adequately control for variability related to subject and stimulation-recording electrode-pairs within subjects.

***Supplementary Figure 4.*** ***Stimulation electrode-type-stratified analysis of CCEP responses across PW conditions.*** Peak Amplitude for AdTech and PMT electrodes across 50 µs, 80 µs, and 120 µs PW conditions.

**Association between Robustness Ratio and Peak Amplitude metrics with Root Mean Square.**

We compared the implemented Robustness Ratio and Peak Amplitude evoked response feature to Root Mean Square (RMS). Specifically, RMS of the average phase (RMSAveragePhase). We calculated RMS by taking the Hilbert transformation of individual stimulation trials, averaging the phase across trials, and then taking the RMS of the average. This measure therefore represents the consistency and strength of phase-locked response across trials. The Spearman rank-based correlation was then calculated for all recording sites between RMS of Average Phase. Strong positive correlations were found for every pulse width condition between Robustness Ratio and RMS of Average Phase (50 µs: 0.954 80 µs: 0.965, 120 µs: 0.966), as well as between RMS with Peak Amplitude (50 µs: 0.463, 80 µs: 0.664 120 µs: 0.627. These results demonstrate the correspondence between Robustness Ratio with more conventional SPES measures and highlight RR as a viable indicator of both strength and consistency of trial-averaged evoked responses.

***Supplementary Figure 5.*** ***Spearman correlations between Root Mean Squared of Average Phase with Robustness Ratio and Peak Amplitude measures.*** The RMS of Average Phase correlation was plotted for each PW condition, and correlations were calculated relative to both Robustness Ratio and Peak Amplitude (n = 811) for stimulation in either the ACC or OFC. Loess fit is overlaid in black.

**Assessing the interaction of Robustness Ratio with Tract Distance between stimulation and recording electrode sites.**

The main manuscript utilizes Euclidean distance as the distance metric for assessing how the nonlinear evoked response pattern interacts with distance of recording from stimulating locations (ipsilateral connections only). However, we additionally evaluated this relationship using path lengths estimated from tractography derived from a normative population. Though patient-specific diffusion neuroimaging was not available for many of our patients, we used data from 10 control subjects collected as a part of the Human Connectome Project (3T Young Adult diffusion MRI database)(Van Essen et al., 2013). Anatomically Constrained Tractography in MRTrix3(Smith et al., 2012) was run and the average streamline length connecting stimulation and recording regions (each recording site was localized to the nearest recording region from the joint HCP-XTRACT atlas). We first investigated the correlation between Euclidean and Tract distances and found a significant positive correlation (Spearman’s rho coefficient = 0.670, *p* < 0.0001) (Panel A). We next repeated the linear mixed effects modeling approach from Figure 3 of the main manuscript using Tract Distance in place of Euclidean distance. We found that results were consistent with those reported in the main manuscript, with Tract Distance being a significant predictor of Robustness Ratio (β = -0.0538 ± 0.00360, *p* < 0.0001), and the rate of decrease in RR is lowest for short PW (β = 0.0162 ± 0.00159 *p* < 0.0001) and moderate for the long PW (β = 8.97 ± 0.00159, *p* < 0.0001) relative to the medium PW (Panel B). We also found that recording sites with the highest RR following 50 µs stimulation displayed significantly different (further) (H = 115.37, *p* < 0.0001) Tract Distances compared to the 80 and 120 µs conditions (80 µs and 50 µs: z = -7.58, *p* < 0.0001; 50 µs and 120 µs: z = -2.55, *p* = 0.0327; 80 µs and 120 µs: z = 8.83, *p* < 0.0001) (Panel C). The distribution of recording channels across sampled Tract distances is also included in Panel C for each PW level.

***Supplementary Figure 6.*** ***Evaluation of Tract Distance as a distance metric*. (A).** Spearman’s rank-based correlation comparing computed Euclidean and Tract distances for each recording site. Both measures are in millimeters. **(B).** Linear mixed effects modeling was used to assess the interaction between Robustness Ratio and Tract Distance from stimulation across each PW condition. **(C).** Recording sites were sorted by PW condition producing the maximal response (in terms of RR) and the median Tract Distance between recording and stimulating locations was compared across preferred PW condition (left). Error bars demonstrate Median Absolute Deviation (MAD). Distributions are displayed as density plots for visualization of Tract Distances across recording electrode sample (right).

**Supplementary Table 4. Brain regions included in region-based analysis of pulse width modulation.** Sampled brain regions were determined using electrode localization, as detailed in the Methods section of the main manuscript. Included brain regions support that at least 1 patient had electrode coverage in that area for detection of ipsilateral connection with the stimulated region.

**Brain Region**

Anterior Cingulate Cortex

Frontal Aslant Tract

Inferior FrontoOccipital Fasciculus

Putamen

Insula and Frontal Opercular Area

Premotor Cortex

Superior Longitudinal Fasciculus 1

Dorsolateral Prefrontal Cortex

Posterior Cingulate Cortex

Cingulum Bundle Dorsal

ParaCentral and MidCingulate Cortex

Superior Thalamic Radiation

Ventromedial Prefrontal Cortex

Forceps Minor

Orbital and Polar Frontal Cortex

Inferior Frontal Cortex

Thalamus

Auditory Association

Inferior Longitudinal Fasciculus

Lateral Temporal Cortex

Uncinate Fasciculus

Superior Longitudinal Fasciculus 3

Anterior Thalamic Radiation

Amygdala

Medial Temporal Cortex

Forceps Major

Optic Radiation

Dorsal Stream Visual

Middle Longitudinal Fasciculus

Superior Parietal Cortex

Inferior Parietal Cortex

Acoustic Radiation

Fornix

Medial Prefrontal Cortex

Corticospinal Tract

Somatosensory and Motor Cortex

Arcuate Fasciculus

**Exploratory analysis of stimulation tissue type across stimulation conditions.**

We performed an exploratory analysis to assess whether pulse width effects differed across stimulation tissue types, given prior reports of tissue-specific stimulation effects. We combined stimulation sites across the ACC and OFC and performed distance-based modeling, calculating the Euclidean distance of each stimulation contact (n = 19) from the gray-white boundary surface. We fit two linear mixed-effects models with random intercepts for stimulation-recording pairs to test how pulse width – amplitude stimulation condition (reference level as 80 µs PW), distance from the gray-white boundary, and Euclidean distance from stimulation site jointly modulate CCEP features (robustness and magnitude).

For Robustness Ratio, strong reductions in respones were found at short and long PW relative to the medium condition (50 µs: *p* < 0.0001; 120 µs: *p* < 0.0001). A strong decay of distance (as seen in the main manuscript) was also detected (β = -0.0732 ± 0.00430, t = -17.1, *p* < 0.0001). No significant effect of distance from the gray-white boundary was detected for the short PW condition, but there was a reversed effect at the long condition (LongPW×Dist2WG: β = −0.183 ± 0.0383, t = −4.78, *p* < 0.0001). Again, as seen earlier, the negative impact of distance on CCEP response was weaker following stimulation at the 50 µs condition (ShortPW×Distance: β = +0.0216 ± 0.00201, t = 10.71, *p* < 0.0001) only.

For Peak Amplitude, strong main effects were found for PW condition (50 µs: β = -12.3 ± 0.95, t = 12.94, *p* < 0.0001; 120 µs: β = -3.94 ± 0.95, t = -4.16, *p* < 0.0001), distance from gray-white boundary (β = -3.14 ± 0.600, t = 5.24, *p* < 0.0001), and distance from stimulation site (β = -0.326 ± 0.0316, t = -10.33, *p* < 0.0001) (Panel A). A white-matter advantageous was observed to be attenuated following stimulation at the 50 µs condition (ShortPW×Dist2WG: β = −2.431 ± 0.364, t = −6.68, *p* < 0.0001) and slightly attenuated at the high condition (LongPW×Dist2WG: β = −0.782, ± 0.364, t = −2.15, *p* = 0.0317). As previously demonstrated in the main manuscript, a decay in signal was reduced at short (Short×Distance: β = 0.143 ± 0.0191, t = 7.47, *p* < 0.0001) but unchanged at the long condition (β = 0.0192 ± 0.0191, t = 1.01, *p* = 0.315). Critically, the white-matter effect diminished with distance (Dist2WG×Distance: β = −0.0423 ± 0.0109, t = −3.88, *p* < 0.0001), yet this erosion was mitigated by positive three-way interactions (ShortPW×Dist2WG×EucDistance: β = +0.0314 ± 0.00661, t = 4.76, *p* < 0.0001; LongPW×Dist2WG×Distance: β = +0.0364 ± 0.00661, t = 5.50, *p* < 0.0001). Overall, these results support that distance between stimulation and recording positions is a dominant factor for both CCEP features and that Peak Amplitude is larger following stimulation in white matter, especially closer to the stimulation site.

***Supplementary Figure 7. Predicted CCEP response (A: RR, B: Peak Amplitude) across Euclidean distance from gray-white (GM/WM) boundary and distance of recording position from the stimulation site****.* Each panel is a heatmap of model-based predictions from modeling. The x-axis represents calculated distance of stimulation site from the gray-white surface boundary (negative values indicate stimulation location deeper in gray matter; positive values indicate deeper in white matter) and the y-axis represents Euclidean distance from the stimulation site. Color exhibits predicted CCEP features, with warmer colors relating to a stronger response. Predictions are based off only fixed effects, while holding random effects constant.

**Exploring the impact of location within Anterior Cingulate Cortex on Evoked Responses.**

Stimulation sites were concentrated in the anterior cingulate cortex (ACC), though precise electrode contact location varied along the rostrocaudal and dorsoventral axes within the ACC. We calculated a weighted score for each stimulation contact, representing the average tendency of recording site preference for PW condition. Scores were normalized to a 0-1 range, where 0 indicated strict preference for the 50 µs condition, 0.5 for the 80 µs condition, and a score of 1 exhibiting sole preference for the 120 µs condition. No visually apparent clusters, pockets, or gradients are evident.

***Supplementary Figure 8.*** ***Weighted score of evoked response preference following stimulation across ACC stimulation sites.*** Response Robustness preference was evaluated across recording electrode contacts for each ACC stimulation site individually. Proportions of preferences across recording sites were calculated and used to compute a normalized score for each stimulation site. Each normalized score was projected onto the glass brain and colored to correspond with PW conditions as presented in the main manuscript (0.0 = 50 µs preference, 0.5 = 80 µs preference, 1.0 = 120 µs preference). Stimulation sites are presented from sagittal, coronal, and axial views to provide maximum anatomical perspectives.

**Supplementary References**

Smith, R.E., Tournier, J.-D., Calamante, F., Connelly, A., 2012. Anatomically-constrained tractography: improved diffusion MRI streamlines tractography through effective use of anatomical information. Neuroimage 62, 1924–1938. https://doi.org/10.1016/j.neuroimage.2012.06.005

Van Essen, D.C., Smith, S.M., Barch, D.M., Behrens, T.E.J., Yacoub, E., Ugurbil, K., 2013. The WU-Minn Human Connectome Project: An Overview. Neuroimage 80, 62–79. https://doi.org/10.1016/j.neuroimage.2013.05.041
